# Supplementary material for: A whole-animal phenotypic drug screen identifies suppressors of atherogenic lipoproteins
Source: eLife. 2026 Aug 27;14:RP105314. doi: 10.7554/eLife.105314 (PMC13521566; doi:10.7554/eLife.105314)
Supplement: Supplementary file 2 [file elife-105314-code1.zip › JHDL_primary_result/Maleic acid/Maleic acid_JHDL_17_019_004_2019_09_20_09_52.pdf]

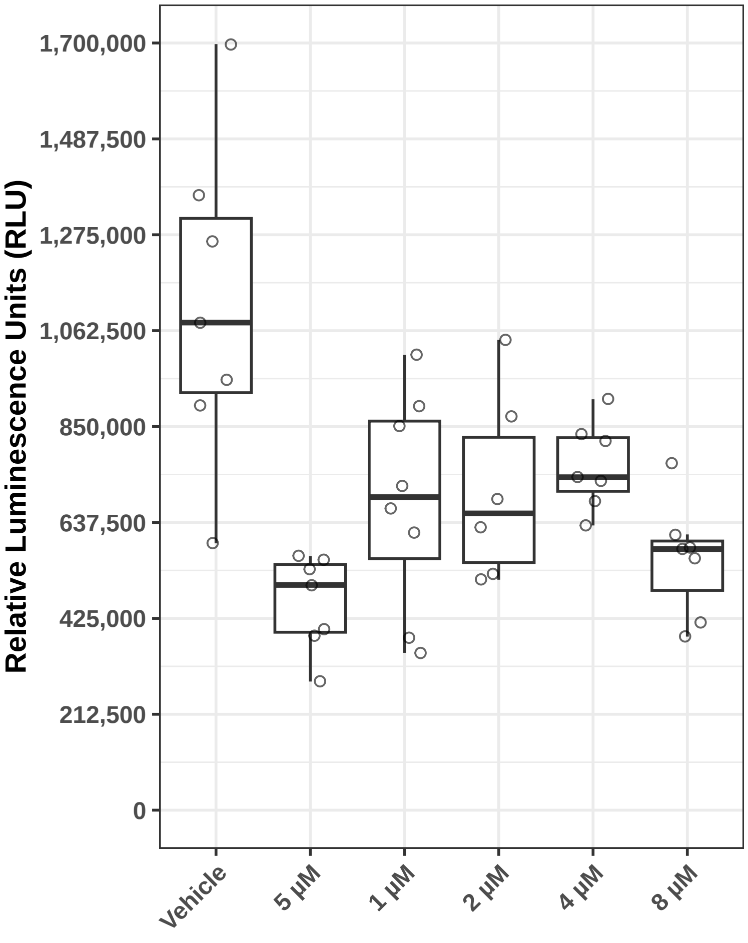

Relative Luminescence Units (RLU)
1,700,000
1,487,500
1,275,000
1,062,500
850,000
637,500
425,000
212,500
0
Vehicle
5 µM
1 µM
2 µM
4 µM
8 µM
